# International cohort study of 73 anti-Ku-positive patients: association of p70/p80 anti-Ku antibodies with joint/bone features and differentiation of disease populations by using principal-components analysis

**DOI:** 10.1186/ar3550

**Published:** 2012-01-06

**Authors:** Katja Lakota, Gerhard G Thallinger, Snezna Sodin-Semrl, Blaz Rozman, Ales Ambrozic, Matija Tomsic, Sonja Praprotnik, Sasa Cucnik, Katjusa Mrak-Poljsak, Angela Ceribelli, Ilaria Cavazzana, Franco Franceschini, Jiri Vencovsky, Laszlo Czirják, Cecilia Varjú, Gunther Steiner, Martin Aringer, Bojana Stamenkovic, Oliver Distler, Marco Matucci-Cerinic, Tanja Kveder

**Affiliations:** 1 Department of Rheumatology, University Medical Centre, Ljubljana, Slovenia; 2 Institute for Genomics and Bioinformatics, Graz University of Technology, Graz, Austria; 3 Rheumatology & Clinical Immunology Unit, Spedali Civili, Brescia, Italy; 4 Institute of Rheumatology, Prague, Czech Republic; 5 Department of Immunology and Rheumatology, Medical School, University of Pécs, Pécs, Hungary; 6 Department of Rheumatology, Internal Medicine III, Medical University of Vienna, Vienna, Austria; 7 Department for Rheumatology, Institute for Treatment and Rehabilitation of Rheumatic and Cardiovascular diseases, Niska Banja, Serbia; 8 Department of BioMedicine, Division of Rheumatology AOUC, Denothe Center, University of Florence, Florence, Italy

## Abstract

**Introduction:**

An international cohort study of 73 anti-Ku-positive patients with different connective tissue diseases was conducted to differentiate the anti-Ku-positive populations of patients based on their autoantibody profile and clinical signs/symptoms and to establish possible correlations between antibodies against Ku p70 and Ku p80 with autoimmune diseases.

**Methods:**

Sera of anti-Ku-positive patients were collected from six European centers and were all secondarily tested (in the reference center); 73 were confirmed as positive. Anti-Ku antibodies were detected with counter-immunoelectrophoresis (CIE), line immunoassay (LIA), and immunoblot analyses. All clinical and laboratory data were follow-up cumulative data, except for anti-Ku antibodies. Statistical analyses were performed by using R (V 2.12.1). The Fisher Exact test was used to evaluate the association between anti-Ku antibodies and diagnosis, gender, clinical signs, and other observed antibodies. The *P *values were adjusted for multiple testing. Separation of disease populations based on the presence of antibodies and clinical signs was investigated by principal-components analysis, which was performed by using thr// R's *prcomp *function with standard parameters.

**Results:**

A 16% higher prevalence of anti-Ku p70 was found over anti-Ku p80 antibodies. In 41 (57%) patients, a combination of both was detected. Five (7%) patients, who were CIE and/or LIA anti-Ku positive, were negative for both subsets, as detected with the immunoblot; 31% of the patients had undifferentiated connective tissue disease (UCTD); 29% had systemic sclerosis (SSc); 18% had systemic lupus erythematosus (SLE); 11% had rheumatoid arthritis; 7% had polymyositis; and 3% had Sjögren syndrome.

**Conclusions:**

A significant positive association was found between female patients with anti-Ku p70 and joint/bone features, and a significant negative association was found between female patients with anti-Ku p80 only and joint/bone features (*P *= 0.05, respectively). By using the first and the third components of the principal-component analysis (PCA) with 29 parameters evaluated, we observed that the anti-Ku-positive population of UCTD patients had overlapping parameters, especially with SLE, as opposed to SSc, which could be helpful in delineating UCTD patients.

## Introduction

The Ku complex is a heterodimer made of p70 and p80 subunits that play major roles in DNA repair, transcriptional regulation, and replication and have also been involved in telomere maintenance, V(D)J recombination, and development of the brain. Ku is ubiquitously found in the nucleus; however, it has also been localized in the cytoplasm, as well as on cellular surfaces [1-3]. In humans, *Ku p70 *and *Ku p80 *genes are localized on different chromosomes (22q13 and 2q33, respectively). Their proteins share sequence homologies, as well as display marked structural homologies. Ku p70 and Ku p80 are generally believed to form and function as a heterodimer, but each also has its own unique activities. Whereas p70 resembles a transcriptional activator, and its DNA-binding domain has been localized to its C-terminus, p80 does not seem to bind DNA and may be involved in interactions with other proteins [4]. The differences also extend to knockout mice models. Ku70-knockout mice showed deficiencies in subgroups of mature T lymphocytes and a significant incidence of thymic lymphoma, whereas Ku p80-knockout mice had arrested T-and B-cell development at early stages and caused growth retardation [5,6].

Although antibodies against Ku antigen (anti-Ku) were originally described in patients with scleroderma-polymyositis overlap syndrome, reports showed that anti-Ku antibodies are found also in many diseases, in particular, in patients with systemic lupus erythematosus (SLE), systemic sclerosis (SSc), and undifferentiated connective tissue disease (UCTD) [7]. The prevalence of anti-Ku antibodies has recently been evaluated in a European EUSTAR-initiated multicenter case-controlled study with 625 SSc patients in whom a clinically distinct subset resulted in 14 anti-Ku-positive patients (2.2%) [8]. The presence of anti-Ku antibodies in the study of Rozman *et al. *[8] was reported to be associated with synovitis, joint contractures, and clinical features of myositis, and negatively associated with vascular manifestation of disease. Cavazzana *et al. *[7] retrospectively analyzed the prevalence and clinical signs of anti-Ku antibodies in patients affected by different autoimmune diseases. Most of anti-Ku-positive patients were found to be affected by UCTD and overlap syndromes, including polymyositis, SSc, and SLE. Interstitial lung disease, myositis, articular symptoms, Raynaud phenomenon, and sicca syndrome represented the main clinical features detected in this cohort. Both recent studies give a broad overview of anti-Ku antibodies and clinical, serologic, and diagnostic correlations.

In 1989, Reeves *et al. *[9] reported on levels of antibodies against Ku p70 and Ku p80 longitudinally over a period of 70 months in sera of patients with SLE (*n *= 2), mixed connective tissue disease (*n *= 1), and Sjögren syndrome (*n *= 1). Their study suggested that anti-Ku p70/anti-Ku p80 antibodies are generated by a selective antigen-driven mechanism; however, polyclonal activation also frequently accompanied autoantibody production. Human Ku autoantibodies are thought to react with at least eight different epitopes of the human complex [10]. SLE sera reacted on immunoblots with at least three epitopes of p70 (aa 560-609, 506-535, and 115-467), and three epitopes of p80 (aa 682-732, 558-681, and 1-374); one strong antigenic region (immunodominant epitope of p70) was revealed near the C-termini of p70 (conformational or discontinuous epitope) and p80 [11].

Yaneva and Arnett [12] examined anti-Ku p70/anti-Ku p80 antibodies with quantitative immunoblotting and found that all positive sera (from mixed ethnic patients with SLE (*n *= 13), SSc (*n *= 9), myositis (*n *= 2), and Sjögren syndrome (*n *= 2)) had antibodies against Ku p80, and only one serum (from a dermatomyositis patient) did not react with Ku p70. Only anti-Sm antibodies appeared to be associated with anti-Ku antibodies.

The aim of our study was to examine whether disease populations, with special emphasis on UCTD, could be depicted based on their associated clinical and laboratory data by using principal-component analysis (PCA). Further, we aimed to investigate the association of anti-p70 and/or anti-p80 antibodies with the presence of clinical signs, autoimmune diseases, and their respective autoantibodies. Therefore, we evaluated the largest European cohort study to date with 73 anti-Ku-positive patients.

## Materials and methods

### Subjects

Sera of anti-Ku-positive patients were collected from six European centers (Ljubljana, Brescia, Pécs, Prague, Nis, and Vienna) and were all secondarily tested; 73 were confirmed as positive in Ljubljana. Among them were 61 female and 12 male subjects. Their mean age was 57.8 years, ranging from 16 to 86 years. Anti-Ku positivity was determined with counter-immunoelectrophoresis (CIE)/line immunoassay (LIA) in the Immunological Laboratory of the Department of Rheumatology, University Medical Center, Ljubljana. Among these, only one patient was LIA but not CIE positive. The diagnoses of SSc, SLE, rheumatoid arthritis, polymyositis, and Sjögren syndrome were defined in the participating centers according to well-established criteria [13-17]. The criteria for UCTD were used, as suggested by Distler *et al. *[18] because this disease has no uniformly accepted diagnostic criteria. The overlaps were gathered under the diagnosis, which represented the leading clinical symptoms and signs of the disease.

Because of the large amount of clinical and laboratory data, besides some single clinical and laboratory features, a more simplified version of presenting the compilation of data was used.

• SLE skin" is defined by the presence of one or more of the following: malar rash, discoid rash, photosensitivity

• Joint and bone" is defined by the presence of one or more of the following: Joint features were evaluated clinically (synovitis and joint contractions) and by radiographic examination (erosive arthritis, acro-osteolysis)

• Muscle" is defined by the presence of one or more of the following: weakness or atrophy of muscles, serum CK elevation, EMG, and muscle biopsy in line with the appropriate criteria for inflammatory myopathy

• Neurology" is defined by the presence of one or more of the following: seizures, psychosis, or peripheral neuropathy

• Gastrointestinal" is defined by the presence of one or more of the following: dysphagia, gastroesophageal reflux, or early satiety

• Pulmonary" is defined by the presence of one or more of the following: dyspnea, NYHA I/II, fibrosis (plain radiograph), restrictive defect, or pleuritis

• Heart" is defined by the presence of one or more of the following: palpitations, conduction blocks, or abnormal diastolic function

• Renal" is defined by the presence of one or more of the following: proteinuria (more than 0.5 g/day), renal insufficiency, or renal crisis

• Antiphospholipid antibodies" are defined by the presence of one or more of the following: anticardiolipin antibodies, or anti-β_2_-glycoprotein antibodies, lupus anticoagulant, detected at least on two occasions, as defined by APS criteria [19]

• Cytopenia" means a low number of leukocytes and/or erythrocytes and/or thrombocytes

All clinical and laboratory data are follow-up cumulative data, except for anti-Ku antibodies, as determined with CIE/LIA and anti-p70/anti-p80 immunoblot detection. Informed consent was obtained, and the study was approved by the Ethics Committee of the Slovenian Ministry of Health.

### Methods

Stored sera were sent on dry ice to the reference center (Department of Rheumatology, Ljubljana), where they were tested. Antibodies against soluble nuclear antigens (anti-ENA): Ro/SS-A, La/SS-B, Sm, U1RNP, PCNA, SL, Scl-70, PM/Scl, Jo-1, and Ku were screened with CIE, as described [20], which show conformational epitopes and are not able to discriminate between anti-Ku p70 and/or anti-Ku p80.

Sera from the aforementioned centers were additionally tested in the same reference center for antinuclear antibodies (ANAs) with indirect immunofluorescence on HEp-2 cell-line substrate (Immunoconcepts, Sacramento, CA, USA) and with LIA for autoantibodies present in myositis, which detects, besides anti-Ku, also antibodies against Jo-1, Mi-2, PM/Scl, and U1-snRNP (Imtec-Myositis-LIA; Imtec, Immunodiagnostika, Berlin, Germany). The Ku antigen on Imtec-Myositis-LIA is a human recombinant Ku p70/p80, which also does not distinguish between the two subunits. Hep-2 patterns were detected with indirect immunofluorescence, rheumatoid factor, with latex fixation test and Waaler Rose reaction, anti-cyclic citrullinated protein antibodies with enzyme-linked immunosorbent assay (ELISA; Immunoscan CCPlus, Euro-Diagnostica AB, Malmö, Sweden), aPL with an in-house ELISA (for anti-aCL IgG and IgM isotypes and for anti-β_2_-GPI, IgG, IgM, and IgA have been measured) and anti-dsDNA antibodies with an in-house FARR-RIA assay.

To detect anti-p70 and anti-p80 antibodies, immunoblotting was used. Nuclear extracts were prepared from THP-1 (human acute monocytic leukemia cell line). Centrifuged THP-1s were washed with PBS, and protease inhibitors (Halt Protease Inhibitor Cocktail Kit; Pierce, Rockford, IL, USA) were added. Extraction was done according to instructions (NE-PER Nuclear and Cytoplasmic Extraction Reagents; Pierce), and protein concentration in nuclear extract was measured with the Bio-Rad Protein Assay (Bio Rad, Munich, Germany). The immunoblot method included an initial immunoprecipitation step, which was performed with Protein A/G PLUS-Agarose Immunoprecipitation Reagent; Santa Cruz, Santa Cruz, CA, USA): 500 μg of proteins from the nuclear extract was mixed with 2 μg mouse monoclonal antibody Ku-3 (clone 162) (NeoMarkers, Fremont, CA) and incubated 2 hours at 4°C. Then 20 μl of resuspended A/G Agarose was added, and the mixture was incubated another 2 hours at 4°C. Washing of pellet was done with PBS for 4 times, each time followed by centrifugation. After the final wash, pellets were resuspended in PBS and electrophoresis SDS-sample buffer and boiled for 3 minutes for protein elution. Sample volume corresponding to 50 μg of primary nuclear extract/cm of gel length was analyzed on 10% SDS-polyacrylamide gels in Tris/glycine buffer in BioRad Mini Protean apparatus and transferred to nitrocellulose (BA 85; Schleicher & Schuell, Munich, Germany) at 100 V, 250 mA for 45 minutes. The dry membrane was blocked with TBS/0.05% Tween buffer with 5% milk. Patient sera were diluted 1:50 and incubated 2 hours, followed by washing and 40 minutes of goat anti-human IgG-HRP (1:1,000) (BioRad) incubation. Detection was done with luminol (Western Blotting Luminol Reagent, Santa Cruz, CA, USA) in G:box (Syngene, Cambridge, UK) with chemiluminescence. As positive controls, monoclonal goat antibodies (Ku-86 (C-20), Santa Cruz; Ku-70 (C-19), Santa Cruz, diluted 1:500) and donkey anti-goat IgG-HRP conjugate (diluted 1:1,000, Santa Cruz) were used. All samples were tested also for colorimetric determination, and the secondary antibodies used were rabbit anti-human alkaline phosphatase (Bio Rad) followed by the substrate NBT/BCIP (Pierce).

### Statistical analyses

Statistical analyses were performed by using R (V 2.12.1) [21]. The Fisher Exact test was used to evaluate the association between anti-Ku antibodies and diagnosis, gender, clinical signs, and other observed antibodies. The *P *values were adjusted for multiple testing by using the approach of Benjamini and Hochberg [22]. An adjusted *P *value below or equal to 0.05 was considered statistically significant. Separation of disease populations based on the presence of antibodies and clinical signs were investigated with PCA. PCA was performed by using the R *prcomp *function with standard parameters. PCA allows the identification of latent variables (principal components) in the data based on observed variables (in our case, 29 parameters, the presence of those autoantibodies and clinical signs that show five or more observations).

## Results

### Anti-Ku p70/80 prevalence

Within our anti-Ku-positive population (*n *= 73), 60 (83%) anti-Ku p70-positive patients were found, whereas 48 (67%) patients were anti-Ku p80 positive. This represents a 16% higher prevalence of anti-Ku p70 over anti-Ku p80. For one UCTD patient, the immunoblot p70/p80 differentiation was not able to be performed. Nineteen (26%) patients had only anti-Ku p70, whereas seven (10%) patients had only anti-Ku p80 antibodies detected (Table 1). In 41 (57%) patients, a combination of both anti-p70/anti-p80 antibodies was seen. Only five (7%) patients who were CIE anti-Ku positive were negative for both subsets of antibodies in the immunoblot (Table 1). Twenty anti-Ku-negative controls (blood donors) were tested only in the immunoblots and did not show any reactivity (data not shown).

**Table 1:** Prevalence of anti-Ku p70/p80 in patient groups as detected with immunoblot analysis

Diagnosis	Number of patients	Ku p70	Ku p80	Ku p70 only	Ku p80 only	Ku p70 and Ku p80	Ku pXX None
**PM/DM**	6	4 (67%)	3 (50%)	2 (33%)	1 (17%)	2 (33%)	1 (17%)
**RA**	8	7 (88%)	6 (75%)	2 (25%)	1 (13%)	5 (63%)	0 (0)
**SLE**	13	10 (77%)	11 (84%)	1 (8%)	2 (15%)	9 (69%)	1 (8%)
**SS**	2	2 (100%)	2 (100%)	0 (0)	0 (0)	2 (100%)	0 (0)
**SSc**	21	17 (81%)	11(52%)	8 (38%)	2 (10%)	9 (43%)	2 (10%)
**UCTD**	22	20 (91%)	15 (68%)	6 (27%)	1 (5%)	14 (64%)	1 (5%)
**Total**	72	60 (83%)	48 (67%)	19 (26%)	7 (10%)	41 (57%)	5 (7%)

Numbers of patients in each group are indicated, followed by the percentage. Ku pXX.none, neither anti-Ku p70 nor anti-Ku p80 present; PM/DM, polymyositis/dermatomyositis; RA, rheumatoid arthritis; SLE, systemic lupus erythematosus; SS, Sjögren syndrome; SSc, systemic sclerosis; UCTD, undifferentiated connective tissue disease.

### Anti-Ku p70/80 association with clinical signs, symptoms, and diagnosis

**Figure 1:**
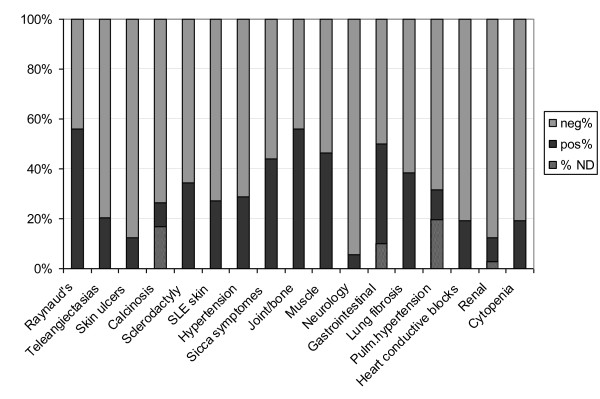
**The number of total patients is 73, except for the columns with not determined (ND) samples**. For a detailed overview of the clinical signs and symptoms, please refer to the Materials and methods Subjects section. ND, the data are not available because of missing procedures necessary to define the particular parameter.

We next analyzed associations with clinical symptoms and presentations. An overview of associations between anti-Ku-positive patients, as determined by CIE/LIA, with clinical signs and symptoms is given in Figure 1. When looking specifically at anti-Ku p70 and/or anti-Ku p80, a significantly positive association between anti-Ku p70 antibodies was exclusively found with joint/bone features (synovitis, erosive arthritis, joint contractures, and acro-osteolysis) (*P *= 0.05), whereas a significant negative correlation was found between anti-Ku p80 antibodies only and joint/bone features, even after adjustment of the *P *value for multiple testing. Both findings were in female patients. No significant associations were found between the presence of anti-Ku p70 and/or anti-Ku p80 antibodies and any of the diagnoses, even when sex or age-related associations were considered.

### Anti-Ku p70/80 association with other autoantibodies

**Figure 2:**
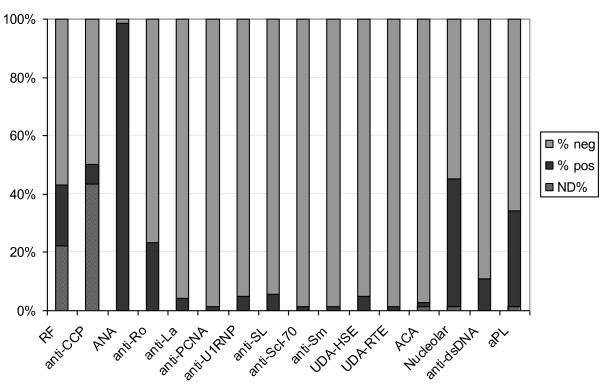
**The total number of patients is 73, except for the columns with not determined (ND) samples**. ACAs, anticentromere antibodies; ANAs, antinuclear antibodies; anti-CCPs, antibodies to cyclic citrullinated peptide; anti-SL (sicca/lupus), anti-Scl-70 (DNA topoisomerase I), anti-dsDNA, antibodies against double stranded DNA; aPLs, antiphospholipid antibodies; ND, data are not available, because of missing procedures necessary to define the particular parameter; PCNA, proliferating cell nuclear antigen; RF, rheumatoid factor; UDA-HSE and UDA-RTE, undefined antibodies as detected with CIE by using human spleen and rabbit thymus, respectively, as sources of antigens.

A summary of the percentage of anti-Ku-positive patients, as determined by CIE/LIA to all autoantibodies measured in this study, is shown in Figure 2. A single anti-Ku positive (positive for both anti-Ku p70 and anti-Ku p80 antibodies) anti-centromere antibodies (ACA)-positive case was found, and this patient was clinically diagnosed as having SLE/Sjögren syndrome. All but one anti-Ku-positive patients were ANA positive, showing mostly a speckled or a speckled plus nucleolar immunofluorescence pattern in all groups.

No significant associations were found between the presence of anti-Ku p70 and/or anti-Ku p80 antibodies and any autoantibodies, when sex- or age-related associations were considered. However, before adjustment of the *P *value, a significant positive association was found between antiphospholipid antibodies and anti-Ku p80 antibodies only, in both the overall and female populations.

### Patient stratification based on presence of clinical signs and antibodies

**Figure 3:**
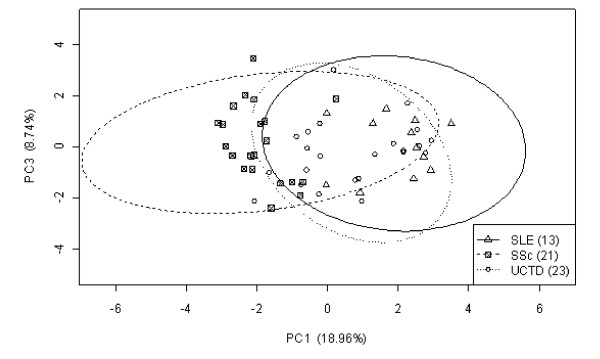
**Principal-component analysis of the presence of 10 antibodies and 19 clinical signs for 57 patients diagnosed with SSc, SLE, or UCTD**. The plot is based on principal components 1 and 3, which explain 18.96% and 8.74% of the total variance of the data, respectively. The individual disease populations are circumscribed by an ellipse representing an 87% confidence level.

To investigate whether it is possible to stratify the patients diagnosed with SLE, UCTD, and SSc based on their autoantibody status and clinical signs/symptoms, we used PCA (Figure 3). The principal components (PCs) are ordered according to the amount of variance in the data they explain, with PC1 being the most informative.

By using PC1 and PC3 for the PCA, we observed that the anti-Ku-positive SLE and SSc populations had little overlap, whereas the anti-Ku-positive UCTD patients in our cohort had a greater overlap with anti-Ku-positive SLE patients than with anti-Ku-positive SSc patients.

## Discussion

A significant association was recently demonstrated between the presence of anti-Ku antibodies and musculoskeletal features in SSc [8]. The current study focuses on the differences between the clinical presentation of anti-Ku p70- and anti-Ku p80-positive patients, regardless of their diagnoses. The study included six European centers, all known for their expertise in the field of systemic autoimmune diseases. With a total of 73 patients, this cohort is the largest to date and showed an overall 16% higher prevalence of antibodies detected against the Ku p70 subunit as compared with Ku p80, which is a novel finding. These data differ from those of a study by Wang *et al. *[23], in which they showed a 28% higher prevalence of autoantibodies against p80 on a total of 58 systemic autoimmune patients; however, this population was examined in the United States and presumably differs from our European cohort. Additionally, the anti-Ku antibodies were detected with ELISA or immunoprecipitation by using K562 cell Ku antigen, and the clinical diagnoses and disease definitions differed from those used in our study, which could have also influenced the results. The same report also indicates the presence of stabilizing autoantibodies (in terms of stabilizing intermolecular contacts between the p70 and p80 Ku subunits) against both recombinant antigens. In the current study, UCTD and SSc patients were the most prevalent in the anti-Ku-positive group, whereas in the Wang *et al. *[21] study, SLE and mixed connective tissue disease overlap patients were highest in number.

Epitope mapping has identified at least eight autoepitopes on the Ku antigen, located on p70, p80, or both subunits, suggesting strongly that an antigenic conformational epitope exists on the C-terminus of p70 and p80 [11]. In our case, five of the patients (7%) who were anti-Ku positive by CIE/LIA were negative for both subsets of antibodies in the immunoblot assay and could be implied to recognize a conformational epitope.

No significant association was found between anti-Ku p70 and/or anti-Ku p80 and any of the diagnoses, even when looking for sex- and/or age-related associations. However, a significant positive correlation (*P *= 0.05) was found between anti-Ku p70 and joint/bone features, and a significantly negative correlation was shown between anti-Ku p80 and joint/bone features (in the female population of anti-Ku-positive patients, representing 84% of the overall population). The correlation is evidently not strong; however, looking at the typical inflammatory joint disease, such as RA, it is worth mentioning the main characteristics of our eight RA patients showing relatively mild joint affliction. The disease activity (by ESR and CRP) was (looking longitudinally) described as high in two patients, medium in two patients, and low in the rest. The radiographic examination of the diseased joints was reported as highly destructive with deformations in one patient and rather mild with slight narrowing of the joint space and few erosions in the rest. The immunoserology showed five seropositive patients (RF-latex and Waaler Rose test and/or anti-CCP-ELISA) and three seronegative patients for both. Furthermore, they typically had high titers of anti-nuclear antibodies. As expected, four patients had anti-Ro, and two patients had anti-La antibodies.

These results might provide the basis for further studies of anti-Ku-related pathogenesis, particularly with respect to joint/bone features. Of interest, Ku p70-knockout mice have increased rates of primary mouse ear fibroblast transformation [24], and embryonic fibroblasts from Ku 80-knockout mice have a prolonged G_2 _phase after H_2_O_2 _treatment [5,25]. It would be interesting to determine the specific roles of the antigens Ku p70/p80 in synovial fibroblasts, especially in DNA-dependent protein kinase catalytic subunit independent processes.

The compilation of specific clinical signs/symptoms and the diagnoses-related prevalence of certain organs/tissues being affected could have introduced a certain bias in the analyses, because of this targeted approach, as described in Materials and Methods. Also, patient diagnoses and clinical signs coming from many different centers and evaluated by different clinicians could result in variations of interpretation, and we do not exclude some possible bias arising from this. However, the representatives from each center were instructed to reevaluate the clinical charts of all patients included. Concerning the CIE/LIA anti-Ku-positive group, the lack of ACA (which had been previously shown [8]) was now confirmed in our larger anti-Ku-positive SSc group. Just one anti-Ku-positive case (with antibodies against both subunits) was ACA positive and diagnosed as having SLE/Sjögren syndrome.

To compare UCTD with SLE and SSc, and to understand the complexity of clinical features in systemic autoimmune diseases, we stratified anti-Ku-positive patients by using PCA. The analysis showed a clear differentiation of anti-Ku-positive SLE and SSc patients, whereas anti-Ku-positive UCTD patients were positioned in between, with a tendency for greater overlap of UCTD with SLE than with SSc. In our Caucasian population of patients, this could imply that the detection of anti-Ku antibodies might help in stratifying UCTD.

## Conclusions

In conclusion, anti-Ku p70/p80 antibodies are not representative markers of a single systemic autoimmune disease. In our study, we showed a higher prevalence of antibodies against Ku p70 as compared with anti-Ku p80. PCA analysis showed that anti-Ku-positive UCTD patients in our cohort had a greater overlap with anti-Ku-positive SLE patients than with SSc patients. Therefore the finding of anti-Ku positivity in UCTD patients might indicate the progression to SLE.

Anti-Ku p70 was positively and anti-Ku p80 was negatively associated (even after adjustment of the *P *value) with joint/bone features, irrespective of diagnosis within connective tissue diseases, which could be an indication of the p70 Ku antigen potential speculative protective role in joint pathophysiology.

## Abbreviations

ACAs: anticentromere antibodies; ANAs: antinuclear antibodies; anti-CCPs: antibodies to cyclic citrullinated peptide; anti-dsDNAs: antibodies against double-stranded DNA; aPLs: antiphospholipid antibodies; anti-Scl-70 (DNA topoisomerase I); anti-SL (sicca/lupus); PCNA: proliferating cell nuclear antigen; PM/DM: polymyositis/dermatomyositis; RA: rheumatoid arthritis; RF: rheumatoid factor; SLE: systemic lupus erythematosus; SS: Sjögren syndrome; SSc: systemic sclerosis; UCTD: undifferentiated connective tissue disease; UDA-HSE and UDA-RTE: undefined antibodies as detected with CIE by using human spleen and rabbit thymus: respectively: as sources of antigens.

## Competing interests

The authors declare that they have no competing interests.

## Authors' contributions

KL was involved in manuscript writing, data generation (immunoblot assays and compilation of the data in table form), and data analysis. GGT performed the statistical analysis, including the principal-component analysis, and was involved in critical data interpretation and manuscript editing. SS-S was involved in study planning and design, interpretation of results, and manuscript writing/editing. BR was in charge of the overall clinical data, their interpretation, and critical review. SC took part in the autoantibody measurements, interpretation of the results, and manuscript writing. KM-P was involved in immunoassays and manuscript drafting. AA, MT, SP, AC, IC, FF, JV, LC, CV, GS, MA, BS, OD, and MM-C all contributed sera samples, clinical data from their centers, and critical manuscript editing and review. TK provided mentorship and was involved in planning of the study and manuscript editing. All authors read and approved the final manuscript.
